# Arboreal and Terrestrial Frugivore Vertebrate Assemblage Diversity and Composition Vary With Disturbance Histories in Guianan Forests

**DOI:** 10.1002/ece3.74374

**Published:** 2026-09-28

**Authors:** Raphaëlle Abensur, Opale Coutant, Pierre Gloaguen, Malika Gottstein, Coraline Pelon, Eric Guilbert, Christophe Baltzinger, Pierre‐Michel Forget

**Affiliations:** ^1^ Muséum National d'Histoire Naturelle, UMR 7179 MECADEV CNRS‐MNHN, LABEX DRIIHM Paris France; ^2^ Field Museum of Natural History Chicago Illinois USA; ^3^ Université Bretagne Sud, UMR 6205 LMBA Lorient France; ^4^ INRAE, EFNO, Domaine des Barres Nogent‐sur‐Vernisson France

**Keywords:** Amazonian mammals and birds, camera trapping, frugivory, plant–animal interactions, seed dispersal, tropical forests

## Abstract

Frugivorous vertebrates, as seed dispersers and predators, play critical roles in forest ecosystem dynamics. Understanding their responses to human disturbances is essential for assessing ecosystem functioning and guiding conservation programs. Using 60 camera traps at nutmeg trees (*Virola kwatae* and *V. michelii*, Myristicaceae), we monitored terrestrial and arboreal frugivores across three forest sites in French Guiana submitted to various human disturbances: a protected mature forest (least disturbed), a formerly exploited forest (intermediately disturbed), and a forest near a road subject to current hunting and logging (most disturbed). We assessed diversity, taxonomic composition and assemblage structure, while comparing visitation rates among sites. Richness remained consistent across all sites, with 19–20 species detected in the canopy and 14–16 in the understory. However, visitation rates significantly varied among sites. Specifically, terrestrial visitation rates decreased as disturbance levels increased (from 232.5 ± 53.7 individuals/100 trap‐days at the protected site to 87.2 ± 11.2 at the most disturbed site). Conversely, arboreal visitation rates tended to increase with increasing disturbances (from 43.5 ± 12.5 at the protected site to 80.3 ± 16 at the most disturbed site). In the canopy, the most disturbed site showed higher visits of kinkajous ( *Potos flavus* ) and golden‐handed tamarins ( *Saguinus midas* ). The understory at this same site had significantly lower visitation rates of “sensitive” species (those with low reproductive rates or targeted by hunters), such as white‐lipped peccaries ( *Tayassu pecari* ) and gray‐winged trumpeters ( *Psophia crepitans* ). In the canopy, site identity, tree diameter, fruit availability and canopy cover were significant predictors of assemblage composition. By contrast, none of the measured micro‐habitat variables contributed to the structure of the terrestrial assemblage, site identity being the single significant predictor. These findings underscore the importance of considering species‐specific responses and site disturbance histories, and highlight the value of protected and restoration areas in maintaining functional assemblages in tropical forests.

## Graphical abstract

This study examined the impact of human disturbances on arboreal and terrestrial fruit‐consuming vertebrate assemblages across three tropical forest sites in French Guiana. While species richness remained consistent across sites, results revealed significant variations in visitation rates and a shift in dominant species. Specifically, the most disturbed area exhibited lower terrestrial frugivore visitation rates and the loss of hunting‐sensitive species, whereas arboreal visits became heavily dominated by kinkajous. These findings underscore the critical role of protected and recovering forests in maintaining the functional communities necessary for tropical ecosystem dynamics.

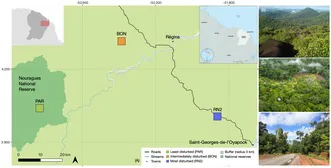

## RÉSUMÉ

Les vertébrés frugivores, en tant que disperseurs ou prédateurs de graines, jouent un rôle essentiel dans la dynamique des écosystèmes forestiers. Étudier leurs réponses aux perturbations humaines est essentiel pour comprendre le fonctionnement global de des écosystèmes et orienter les programmes de conservation. En utilisant 60 pièges photographiques installés au sol et en canopée d'arbres de la famille des Myristicaceae (*Virola kwatae* et *V.michelii*), nous avons suivi les frugivores terrestres et arboricoles sur trois sites forestiers en Guyane (française), soumis à différents niveaux d'anthropisation: une forêt mature protégée (la moins perturbée), une forêt anciennement exploitée (perturbation intermédiaire), et une forêt adjacente à une route faisant l'objet de perturbations fréquentes de chasse et d'exploitation forestière (la plus perturbée). Nous avons évalué la diversité, composition taxonomique et structure des assemblages de frugivores, tout en comparant les taux de visites des arbres entre les sites. La richesse spécifique des consommateurs de fruits était constante sur l'ensemble des sites (19 à 20 espèces en canopée; 14 à 16 en sous‐bois), mais les taux de visite montraient des variations entre les sites. Plus précisément, les taux de visite des frugivores terrestres diminuaient avec l'augmentation du niveau de perturbations (site protégé 232,5 ± 53,7 individus/100 jours‐pièges vs. site le plus perturbé 87,2 ± 11). À l'inverse, les taux de visite des arboricoles avaient tendance à augmenter avec le niveau de perturbations (site protégé 43,5 ± 12,5 vs. site le plus perturbé 80,3 ± 16). En canopée, le site le plus perturbé montrait des taux de visites plus élevés de kinkajous ( *Potos flavus* ) et de tamarins à mains dorées ( *Saguinus midas* ). En sous‐bois de ce même site, les taux de visites étaient significativement plus faibles pour les espèces dites « sensibles » (à faibles taux de reproduction ou ciblées par les chasseurs), comme le pécari à lèvres blanches ( *Tayassu pecari* ) et l'agami trompette ( *Psophia crepitans* ). Par ailleurs, en canopée, l'identité du site, le diamètre de l'arbre, la disponibilité en fruits et le couvert forestier étaient des prédicteurs significatifs de la composition des assemblages. À l'inverse, aucune des variables de micro‐habitat mesurées ne contribuait de manière significative à la structure des assemblages terrestres, l'identité du site étant le seul prédicteur significatif pour l'assemblage des frugivores terrestres. Ces conclusions soulignent l'importance de prendre en compte les réponses spécifiques des espèces et l'historique de perturbations des sites. Elles mettent également en avant la valeur des aires protégées ou en cours de restauration pour le maintien d'assemblages fonctionnels dans les forêts tropicales.

## 1 Introduction

In tropical forests, the majority of the tree species rely on animal‐mediated seed dispersal (Jordano 2000; Boissier et al. 2020; Fricke et al. 2024). Consequently, frugivorous vertebrates play a crucial role in seed dispersal, acting as primary drivers of forest regeneration and floristic diversity maintenance (Ahumada et al. 2011; Boron et al. 2019; Haysom 2022). Effective seed dispersal is fundamental for seedlings to escape density‐dependent mortality near parent trees (Janzen 1970; Connell 1971; Comita et al. 2014). However, tropical ecosystems are impacted by increasing human disturbances driven by rising human demands (Laurance et al. 2012; Boissier et al. 2020). Such pressures threaten the stability of plant–animal interactions by reshaping the structure and composition of plant and frugivore communities (Gardner et al. 2019). These compositional shifts, including species loss and taxonomic turnover, disrupt mutualistic networks and compromise long‐term forest resilience (Clements et al. 2014; Chen et al. 2019). Beyond taxonomic changes, human disturbances also reshape how species use space and resources available. In disturbed environments, the reduction in food availability or increased threats trigger “niche compression”, leading frugivores to concentrate their foraging activity on a restricted number of high‐quality resource patches (MacArthur and Wilson 1967; Manlick and Pauli 2020; Sévêque et al. 2020). Monitoring these interactions is therefore essential to assess the resilience potential of ecosystems facing increasing human disturbances.

In Neotropical forests in particular, human disturbances are increasingly reconfiguring frugivore assemblages through defaunation, often leading to the dominance of specific trophic guilds in defaunated areas that modifies forest's landscapes: for instance, a higher frequency of herbivores and seed predators increases the mortality of seeds, seedlings, and saplings, thereby slowing down forest regeneration (Pires and Galetti 2023). Moreover, the decline of large‐bodied frugivores negatively impacts the dispersal of large‐seeded plants (Béllo Carvalho et al. 2023).

Nutmeg trees are known to be important ecological resources for animal communities, as they provide high‐energy lipids that are essential for a diverse range of frugivores in South America (Howe and Vande Kerckhove 1981; van Roosmalen 1985; Sabatier 1997; Holbrook and Loiselle 2009; Ratiarison and Forget 2013). They are highly dependent on frugivores and have been used as models to study seed dispersal (Howe et al. 1985; Russo 2005; Kitamura and Poonswad 2013).

While a decline in nutmeg fruit removal rates and terrestrial frugivore's abundance at disturbed sites is documented (Holbrook and Loiselle 2009; Boissier et al. 2014, 2020), the impact of disturbances on arboreal frugivore communities remains poorly studied and is often limited to a few diurnal, emblematic species (Moore et al. 2021). Our study contributes to addressing this gap by examining the diversity and composition of frugivores' assemblages in the canopy and understory of forest sites subject to increasing human disturbances.

In the Guiana Shield, forests are being threatened by infrastructure development leading to greater resource exploitation (i.e., hunting, logging, gold‐mining) and habitat fragmentation (Trombulak and Frissell 2000; Laurance et al. 2012; Boissier et al. 2020; Richard‐Hansen et al. 2019). In French Guiana, such extraction activities can affect vertebrate community structure by reducing abundance, specific and functional diversity (Richard‐Hansen et al. 2019; Boissier et al. 2020). These pressures often coexist along a gradient of human accessibility. In this study, we characterize this gradient through three distinct levels of disturbances: (1) a mature, well‐preserved forest with minimal human impact, (2) a formerly exploited site with intermediate disturbance levels, and (3) a forest site located near a major road, where tree logging is combined with increased hunting accessibility. Such disturbance levels create complex filters for frugivores across vertical strata, with potential impacts on seed dispersal and forest regeneration.

Human disturbances can affect arboreal and terrestrial animals differently: arboreal mammal communities, for instance, tend to be more sensitive to habitat fragmentation and forest cover loss than terrestrial ones (Whitworth et al. 2019; Scabin and Peres 2021). In particular, large‐bodied arboreal species, which serve as major seed dispersers, exhibit the strongest negative responses to habitat fragmentation due to their high dependency on canopy cover and tree connectivity (Gregory et al. 2017). In contrast, hunting remains a primary pressure on terrestrial fauna, large rodents and ungulates being the most harvested in French Guiana (Korysko 2019; Osuri et al. 2020).

While quantifying seed dispersal effectiveness is often logistically impossible due to the complexity of tracking seed fates, visitation rates serve as a reliable proxy for quantifying seed dispersal (Jordano 2000; Schupp et al. 2010). Because the frequency of frugivore visits is strongly correlated with the total number of seeds removed, camera traps provide an effective method to capture these interactions (Markl et al. 2012). For *Virola* species, which produce large, nutrient‐rich seeds, the arrival of a potential disperser—detectable via camera traps in both the canopy and understory—represents the primary limiting behavior in the dispersal process. The aim of the study is to characterize arboreal and terrestrial frugivore assemblages visiting *Virola* trees along a forest disturbance gradient in French Guiana. We addressed the following research questions:

- How do human disturbances affect the diversity and composition of arboreal and terrestrial vertebrate frugivore assemblages visiting *Virola* trees?
- Which species contribute the most to the differences observed among sites, for each stratum?
- Which environmental factors help explain species visitation rates?

We hypothesized that human disturbances (e.g., hunting and habitat degradation) act as filters that selectively remove sensitive species—those with low reproductive rates and/or targeted by hunters—and modify assemblage structure.

From this, we derived the following predictions:

1. Diversity indices will decrease with increasing disturbance levels in both the canopy and the understory.
2. Assemblage composition will vary both among sites and between strata. We expect lower visitation rates of hunting‐sensitive species, particularly large‐bodied frugivores with slow life histories, at the most disturbed sites, in both the canopy and the understory.
3. We expect an effect of local environmental factors (i.e., fruit availability, tree species, elevation, canopy cover) on visitation rates. Specifically, we expect the presence of fruits to have a positive effect on arboreal and terrestrial visitors, and low canopy cover to have a negative effect on arboreal visitors.

## 2 Methods

### 2.1 Study Sites and Disturbance Levels

French Guiana, located in northern South America, is predominantly covered by tropical forest, which accounts for about 97% of the land area, including 44% under protected status (Rahm et al. 2021). The Guianan forest is characterized by diverse habitats that host around 1800–2200 tree species, up to 205 mammal species (excluding marine mammals), and 2200 bird species (Sabatier 1990; De Thoisy et al. 2010; Direction Générale des Territoires et de la Mer [DGTM] Guyane 2021; Molino et al. 2022; Marcon et al. 2024). The area has a humid equatorial climate with a stable temperature (mean 26°C **±** 2°C), but exhibits strong seasonal variations in rainfall, with a wet season from December to July and a dry season from August to November (Météo France 2025; Guitet, Brunaux, et al. 2015; Guitet, Pélissier, et al. 2015; Charles‐Dominique et al. 1981). Rainfall patterns act as the primary drivers of tree phenology (Mendoza et al. 2018; Forget and Norwood 2025).

We selected three high‐plateau forest sites in French Guiana representing different levels of human disturbances: (1) a protected mature forest (least disturbed), (2) a formerly mined and logged forest (intermediately disturbed), and (3) a forest adjacent to a national road undergoing active logging and hunting (most disturbed). This classification is based on a combination of forest accessibility, distance to urban centers, and hunting pressure (Table 1) (Brocardo et al. 2023; Benítez‐López et al. 2017). The map of the study sites was edited on QGIS (version 3.26.3‐Buenos Aires) (Figure 1).

**TABLE 1:** Study sites and environmental conditions.

	Nouragues Reserve, Pararé	Bonaventure	National Road 2
Code	PAR	BON	RN2
Status	National reserve	Eco‐tourism camp	State forest
Size (ha)^a^	50.9	10.5	170.5
Elevation ranges (m)	90–200	90–260	55–100
Canopy cover ranges (%)	97.4–99.5	95.8–99.0	86.5–99.5
Disturbance level	Least disturbed	Intermediately disturbed	Most disturbed
Main human disturbances	Scientific research	Former logging and gold mining	Road, hunting, cattle farming, tree logging
Accessibility	By river/helicopter	By track access	By road
Distance to the main city (km)^b^	100	35	20
Hunting status	Prohibited	Allowed	Allowed

^a^ Measured on R with chull function, the surface of the smallest area containing all the camera traps.

^b^ Length of access paths mapped using Google Earth (2026).

**FIGURE 1:**
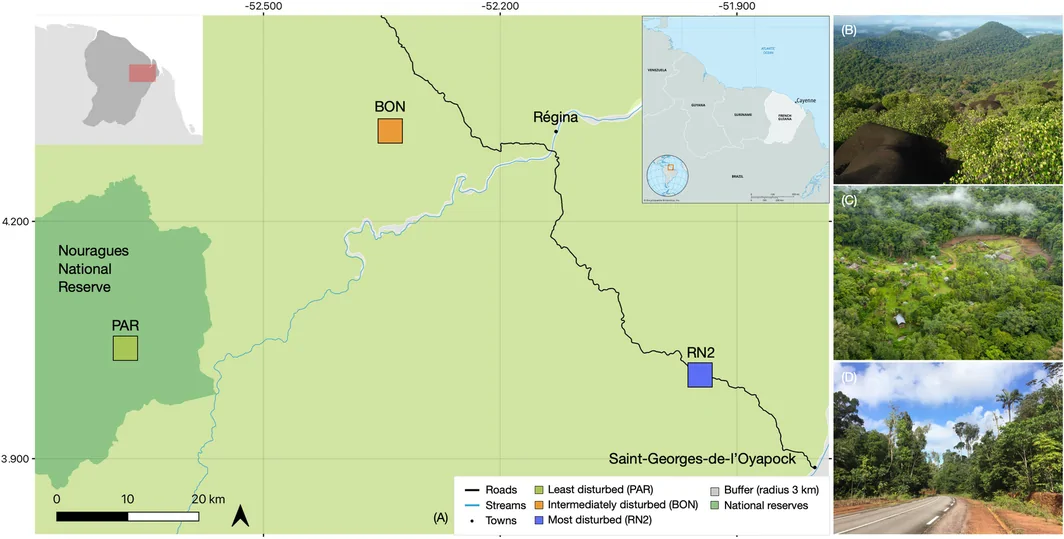
Study areas. (A) Location of the study sites, French Guiana, South America, edited on QGIS (version 3.26.3‐Buenos Aires). (B) Nouragues National Reserve P.‐M. Forget. (C) Bonaventure camp Ye Films. (D) National 2 Road (RN2) P.‐M. Forget.

#### 2.1.1 Least Disturbed Site: Protected Mature Forest

The site is located inside the Nouragues National Reserve at Saut Pararé (hereafter, PAR) (Figure 1). This mature forest is located near the field research station of the Centre National de la Recherche Scientifique (CNRS) and is characterized by minimal human disturbances for scientific research purposes (Bongers et al. 2001). Hunting is forbidden (GéoGuyane 2017). This site is located 100 km upriver from the nearest settlement Régina (1658 inhabitants in 2023; Insee 2026), and only accessible after 4 to 8 h of pirogue depending on the water level, or by helicopter, which strongly limits human presence. The forest exhibits a multilayered structure, with palms reaching up to 5 m, a subcanopy of trees around 10 m tall, a middle canopy, and an upper canopy exceeding 30 m in height.

#### 2.1.2 Intermediately Disturbed Site: Formerly Mined and Logged Forest

The Bélizon forest near the Bonaventure camp (hereafter, BON) was previously logged and gold mined until operations ceased in the early 2000s (ONF 2010). It is currently an eco‐tourism area with relatively small hosting capacity (about 500 visits per year), and casual visitors oriented toward nature‐based tourism (Bonaventure camp managers, pers. comm.). This site represents an intermediate level of disturbances. Restoration efforts (e.g., creek rehabilitation) and natural succession allowed the growth of an advanced secondary forest characterized by the presence of mature trees (ONF 2010). Hunting is not specifically managed in the Bélizon forest but remains scarce due to limited accessibility—with a 10 km‐long track access and 25 km of road from Régina, representing about 1 h by car (GéoGuyane 2017; Google Earth 2026; R. Abensur pers. obs.). Forest structure remains similar to the least disturbed site (PAR).

#### 2.1.3 Most Disturbed Site: Forest Adjacent to the Road

This study site is located along the National Road 2 (hereafter, RN2), between the towns of Régina and Saint‐Georges‐de‐l'Oyapock (4915 inhabitants in 2023; Insee 2026). It represents the most disturbed condition in our study due to high accessibility and active logging and hunting. The construction of new sections of the RN2 in the mid 1990's, aiming at connecting Cayenne to the Brazilian border, significantly increased accessibility to previously remote forest areas. Since the opening to traffic in 2003, this infrastructure development has led to chronic and intensified human impacts on the surrounding natural ecosystems (Coutant et al. 2022). Hunting in this area is officially regulated by departmental quotas (Direction Générale des Territoires et de la Mer [DGTM] Guyane 2011; GéoGuyane 2017). Additionally, since November 2024, the site has undergone legal selective logging managed by the National Forest Office (ONF 2007). This activity involved the harvest of high‐value timber species, primarily *Dicorynia guianensis* (Fabaceae; vernacular name Angélique) and *Qualea rosea* (Vochysiaceae; vernacular name Gonfolo rose), leading to the creation of skid trails and significant canopy openings. These local modifications of the forest cover and landscape structure are expected to influence local animal communities. This forest is characterized by its high accessibility and can be reached from Saint‐Georges‐de‐l'Oyapock in approximately 20 min by car. The forest exhibits a more open structure with palm trees reaching up to 5 m, a tree layer around 10 m tall, with little intermediate vegetation and a high canopy exceeding 30 m in height.

### 2.2 Studied Trees

We selected two nutmeg tree species *Virola kwatae* and *Virola michelii* (hereafter *Virola* spp.). The genus *Virola* (Myristicaceae, vernacular name yayamadou) comprises approximately 60 species of dioecious, annually fruiting trees, representing the most diverse genus of Myristicaceae in America (Santamaría‐Aguilar et al. 2019). These trees produce fleshy fruits that dehisce into two valves when ripe, displaying a single large seed covered by a colored aril which attracts a variety of frugivores (Figure 2A; Sabatier 1997; Santamaría‐Aguilar et al. 2019). Given its role as a key resource provider, its ecological importance has been widely documented (Forget et al. 2001; Ratiarison and Forget 2013; Boissier et al. 2014). The studied species *Virola kwatae* and *V. michelii* coexist at low densities in French Guiana (Fleury et al. 2012; Ratiarison and Forget 2013; Binelli et al. 2020). *Virola kwatae* grows at low density (e.g., 3.4 ind·ha^−1^ at Arataye, Saut Pararé, Sabatier 1997), almost exclusively on slopes, hilltops, and ridges (at elevations of approximately 80–100 m) and can reach a height of 40–50 m (Figure 2B; Séguigne et al. 2022). *Virola michelii* is more common in lowland coastal forests and reaches 30–40 m in height. The size of the seeds differ between species, seeds of *V. kwatae* are slightly bigger (average 2.8 × 1.8 cm) than seed of *V. michelii* (average 2.0 × 1.4 cm), however both species show similar aril to seed ratio (Figure 2C; Ratiarison and Forget 2013; Boissier et al. 2014). As animal‐dispersed species, these trees are highly dependent on frugivores communities (Sabatier 1997; Boissier et al. 2014).

**FIGURE 2:**
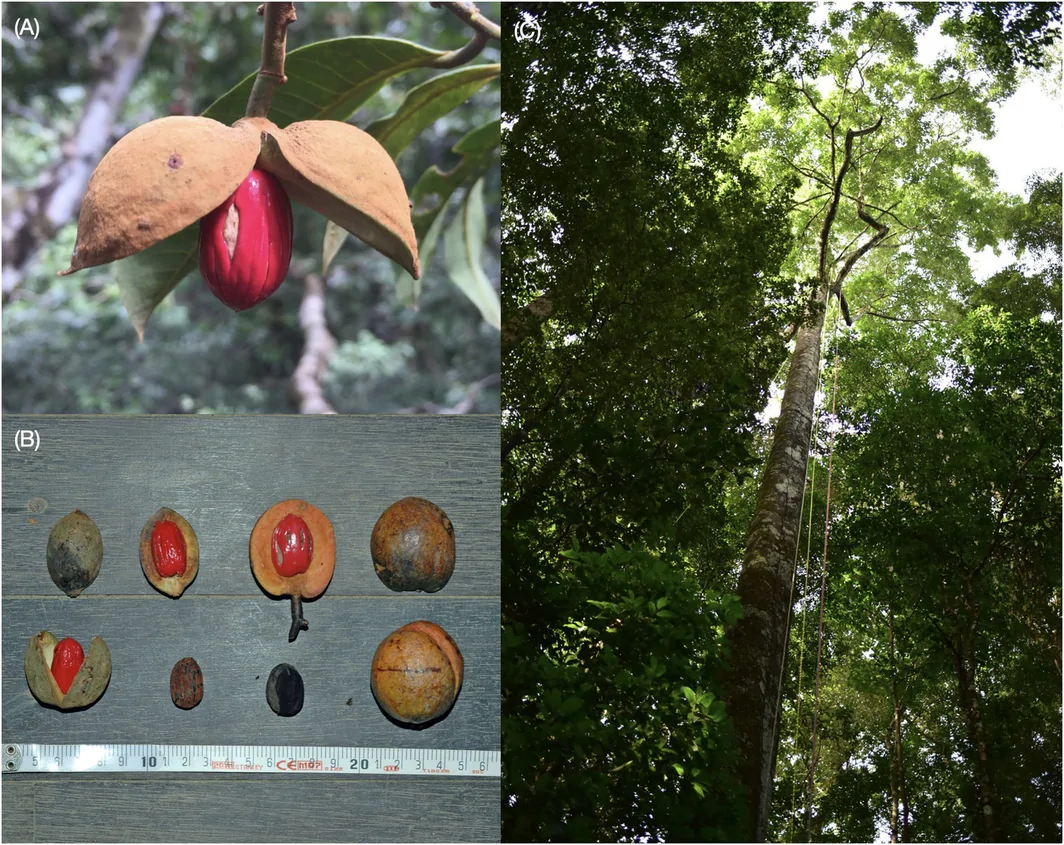
*Virola* fruits and tree. (A) Opened *Virola michelii* fruit. (B) Fruits and seeds of *V. michelii* (left) and *V. kwatae* (right). (C) *Virola kwatae* (A, B: P.‐M. Forget, C: E. Guilbert).

### 2.3 Studied Animals

*Virola* fruits are consumed by arboreal and terrestrial frugivores, which determine the fate of the seeds either through predation or dispersal (Howe 1981; Sabatier 1997; Feer et al. 2001). Consumers play different roles based on their feeding behavior and masticatory abilities: while species that ingest intact seeds or exhibit scatter‐hoarding behavior promote dispersal, seed predators limit recruitment by destroying the seeds during and after ingestion (Feer et al. 2001). A significant proportion of seeds is dispersed by arboreal consumers, primary the red‐faced spider monkey ( *Ateles paniscus* , ~50%) and toucans (mainly *Ramphastos tucanus* and *Ramphastos vitellinus* , ~13%) (Sabatier 1997). Other primates including red‐howler monkeys ( *Alouatta macconnelli* ), and brown and tufted capuchins ( *Sapajus apella* and *Cebus olivaceus* ) also consume and disperse *Virola*. Other arboreal vertebrates such as rodents (i.e., white‐faced spiny rats ( *Echimys chrysurus* ), squirrels ( *Sciurus aestuans* and *Sciurillus pusillus* )) or kinkajous ( *Potos flavus* ) contribute to the seed dispersal of *Virola* trees (Sabatier 1997). The remaining seeds (~30%) fall on the ground and are consumed by terrestrial frugivores (Sabatier 1997; Ratiarison and Forget 2013; Santamaría‐Aguilar et al. 2019). About 40% of these fallen seeds have been shown to be destroyed by terrestrial seed predators, including peccaries (the white‐lipped peccary ( *Tayassu pecari* ) and the collared peccary ( *Pecari tajacu* )), and terrestrial birds such as tinamous (the great tinamou ( *Tinamus major* ) and the variegated tinamou ( *Crypturellus variegatus* )) and black curassows ( *Crax alector* ) (Sabatier 1997). Brocket deers ( *Mazama americana* and *Passalites nemorivagus*) are known to destroy *Virola* seeds during mastication, although their overall ecological role remains undetermined (Gayot et al. 2004). In contrast, the remaining seeds (~60%) are either partially consumed or dispersed by scatter‐hoarding rodents, such as red‐rumped agoutis ( *Dasyprocta leporina* ) and red acouchis ( *Myoprocta acouchy* ), which use small caches that later facilitate germination (Sabatier 1997; Dubost and Henry 2006; Forget and Cuijpers 2008). Among terrestrial birds, gray‐winged trumpeters ( *Psophia crepitans* ) also significantly contribute to seed dispersal (Erard et al. 1991).

### 2.4 Data Collection

#### 2.4.1 Camera Trap Set Up

We collected images with Reconyx camera traps (model HF2X) equipped with infrared detectors and 32 Go memory cards. After prospecting for *Virola* fruiting trees, we equipped a total of 30 trees (10 per study site). Trees were selected based on their accessibility and indicators of fruiting activity, such as visible fruits in the canopy or visible fruits on the forest floor. We equipped each tree with two camera traps, one in the understory and one in the canopy to respectively capture ground‐dwelling and arboreal frugivorous vertebrates. The understory cameras were set up on average from 5 to 10 m from the base of the trees, at a height of 0.5 m, with the lens oriented horizontally toward the fruiting tree to optimize detections of target animals. This height was chosen as a strategic compromise to optimize detections within a multi‐taxa frugivore assemblage, ranging from small rodents to large ungulates (Palencia et al. 2022). The arboreal camera traps were set up in the crowns of the fruiting trees using the single‐rope climbing technique, at heights ranging from 23 to 45 m, with a mean height of 35 m (Table 1). Each camera was set up with the lens facing a branch, and any leaves close to the camera lens were removed to limit false triggers.

The ratio *V. kwatae*/*V. michelii* varied among sites, constrained by the difficulty in finding the target trees. However, we accounted for tree species identity in the analyses (Table 2). The Average Nearest Neighbor (ANN) distance between two cameras was 175 m but varied because the camera placement was influenced by both the presence of target trees and forest accessibility (Table 2). The size of the sampled area on each study site was measured in R with the chull function, representing the surface of the smallest area containing all the camera traps (Figure 1). The distance to the first settlement was measured as the length of access paths mapped using Google Earth (2026).

**TABLE 2:** Sampling effort in each study site, detected independent events (≥ 30 min) total and averaged per trap/day, counts of individuals (includes all individuals either alone or in groups), and frugivores taxonomic richness, after removal of unidentified rodents and birds.

Study site	*N*	Tree species		ANN (m)	Cumulated trap‐days		Independent events		Count of individuals		Ind. events/trap‐days		Recorded richness	
					C	U	C	U	C	U	C	U	C	U
Least disturbed (PAR)	10	*V. kwatae*	9	159	1760	1743	763	3085	791	4061	0.44	1.77	22	16
		*V. michelii*	1											
Intermediately disturbed (BON)	10	*V. kwatae*	4	70	1673	1734	940	2010	984	2661	0.56	1.16	21	18
		*V. michelii*	6											
Most disturbed (RN2)	10	*V. kwatae*	7	298	1599	1630	1212	1112	1328	1415	0.76	0.68	22	16
		*V. michelii*	3											
Total	30				5032	5107	2915	6207	3103	8137				

Abbreviations: ANN = Average Nearest Neighbor distance between two cameras, C = canopy, *N* = number of camera traps per study site, U = understory.

We analyzed the data collected during the wettest season (defined from December 01 to May 31 based on meteorological data), to cover the typical fruiting period of the target trees (Sabatier 1997; Mendoza et al. 2018; Météo France 2025). The cameras were programmed to take five photos when triggered with the “RapidFire” setting for the picture interval, and no delay between triggers. We set the detector to the highest sensitivity. Once installed, the cameras remained operational continuously during the study period (Table 2).

To account for potential spatial autocorrelation arising from this sampling design, we assessed the independence of our sampling units. Specifically, we conducted Mantel tests for each study site and stratum combination to test the correlation between biological dissimilarity (taxonomic composition) and geographic (Euclidean) distance between camera locations. No significant correlation was detected except for BON‐canopy (*r* = 0.362, *p* = 0.043), indicating an absence or weak spatial autocorrelation in our biological data (Table S1).

#### 2.4.2 Human Disturbance Level Assessment

To establish the disturbance levels described above (Section 2.1), we combined qualitative and quantitative proxies. We defined forest accessibility as the way to access the forest: easy (road access), intermediate (track access) and hard (access by river or helicopter).

The distance to the main urban center was the average distance between the camera traps and the nearest settlement, measured by tracing access paths (river, road, or track) on Google Earth (2026) (Table 1). We used the distance to first settlements and accessibility as the main predictors for hunting pressure (Benítez‐López et al. 2017). Forest cover loss in the last 20 years (2001–2022) within a 3‐km radius was initially calculated (Hansen et al. 2013; Global Forest Watch 2026) but subsequently excluded from the analysis, as it was negligible across all sites (< 0.05%) and failed to capture recent local logging.

#### 2.4.3 Environmental Covariates

##### 2.4.3.1 Fruit Availability per Tree

To assess how resource availability influenced species observations, we monitored the fructification status of each focal tree through surveys of crown fruit presence and the presence of fallen fruits on the ground. These observations were obtained from the arboreal camera traps that allowed to track fruit development and maturation per month throughout the study period. Fruit availability was recorded as a binary variable (1 = fruits observed; 0 = no fruit observed).

##### 2.4.3.2 Local Habitat Structure

To characterize local habitat structure, we measured tree density (number of trees with a diameter at breast height (DBH) > 30 cm within a 10 m radius around the focal tree), DBH of each equipped tree, canopy cover using a spherical densiometer and elevation using Google Earth (2026) at each camera location.

##### 2.4.3.3 Seasonal Covariate

To account for the influence of climatic conditions on animal activity, we included rainfall as a temporal covariate in our models. We used monthly rainfall data (mm) as a proxy for regional climatic conditions applied at each study site. Data for the least disturbed site were obtained from the Nouragues Research Station, while data for the intermediately and most disturbed sites were retrieved from the nearest Météo France regional stations (Météo France 2025).

### 2.5 Data Analyses

#### 2.5.1 Data Extraction and Species Identification

The raw images on the camera trap memory cards were uploaded to Agouti.eu, an application for standardized processing of camera trap images (Figure 3; Casaer et al. 2019; GUYACAM Project 2026a). All individuals were identified to the genus or species level using a combination of automatic annotation (Agouti AI) and manual identification by 41 volunteers through a citizen science project, with continuous quality control by taxonomic experts to ensure a fully harmonized dataset (GUYACAM Project 2026b).

**FIGURE 3:**
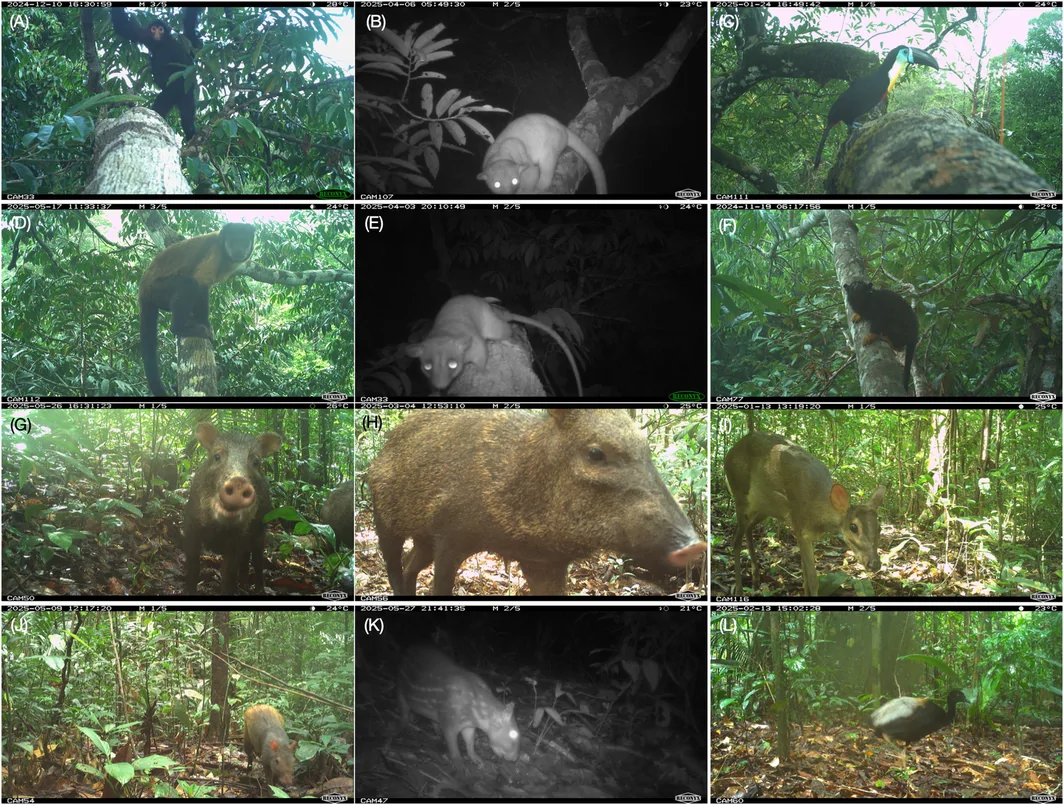
Camera trap pictures of main arboreal (A–F) and terrestrial fruit‐consumer vertebrates (G–L). (A) Red‐faced spider monkey. (B) Kinkajou. (C) Channel‐billed toucan. (D) Brown capuchin. (E) Bare‐tailed wooly opossum. (F) Golden‐handed tamarin. (G) White‐lipped peccary. (H) Collared peccary. (I) Brocket deer. (J) Red‐rumped agouti. (K) Lowland paca. (L) Gray‐winged trumpeter.

We used R (version 4.3.1) with the Camtraptor package to organize the data extracted from Agouti.eu (GUYACAM Project 2026a; Oldoni and Desmet 2022). We defined an independent interaction event as a photo sequence of a species separated by at least 30 min from the next record of the same species at the same sampling point: the optimal independence interval was set at 30 min after identifying the inflection point of normalized species occurrence numbers weighted by camera deployment duration (Relative Abundance Index, hereafter RAI) curves plotted across a range of increasing time thresholds (Yasuda 2004; De Harlez 2023). Thus, we characterized this 30 min detection period as the time from when an individual enters the vicinity of *Virola* until it leaves it. Species that could not be reliably distinguished from images were aggregated into broader taxonomic groups; hereafter, the term “species” refers to both species and aggregated species groups (Emmons and Feer 1997; Gayot et al. 2004). We selected frugivorous animals, defined as species that subsist preferentially on fruit and/or seeds (Smythe 1986). We also included less specialized species for which fruit constitutes at least one‐quarter of their diet, as they maintain non‐negligible ecological interactions through seed dispersal or predation. All observations of non‐fruit‐consumer species were excluded from subsequent analyses (Emmons and Feer 1997; Bongers et al. 2001; the complete list of recorded *Virola* fruit‐consumer species is available in Table S2).

#### 2.5.2 Statistical Analyses

##### 2.5.2.1 Comparison of Diversity Indices

To quantify and compare taxonomic diversity across sites and strata, we generated Hill numbers curves using the iNEXT package, based on a rarefaction and extrapolation approach to standardize for varying sampling effort across study sites (Chao et al. 2014; Hsieh et al. 2016; Bowler et al. 2017). We calculated three indices based on the diversity order *q*: (i) species richness (*q* = 0); (ii) Shannon diversity (*q* = 1), which weights species by their relative abundance; and (iii) Simpson diversity (*q* = 2), the inverse of Simpson's index, which emphasizes dominant species (Figure 4). We defined a sampling unit as one camera trap‐day (Gotelli and Colwell 2001; Si et al. 2014; Haysom 2022).

**FIGURE 4:**
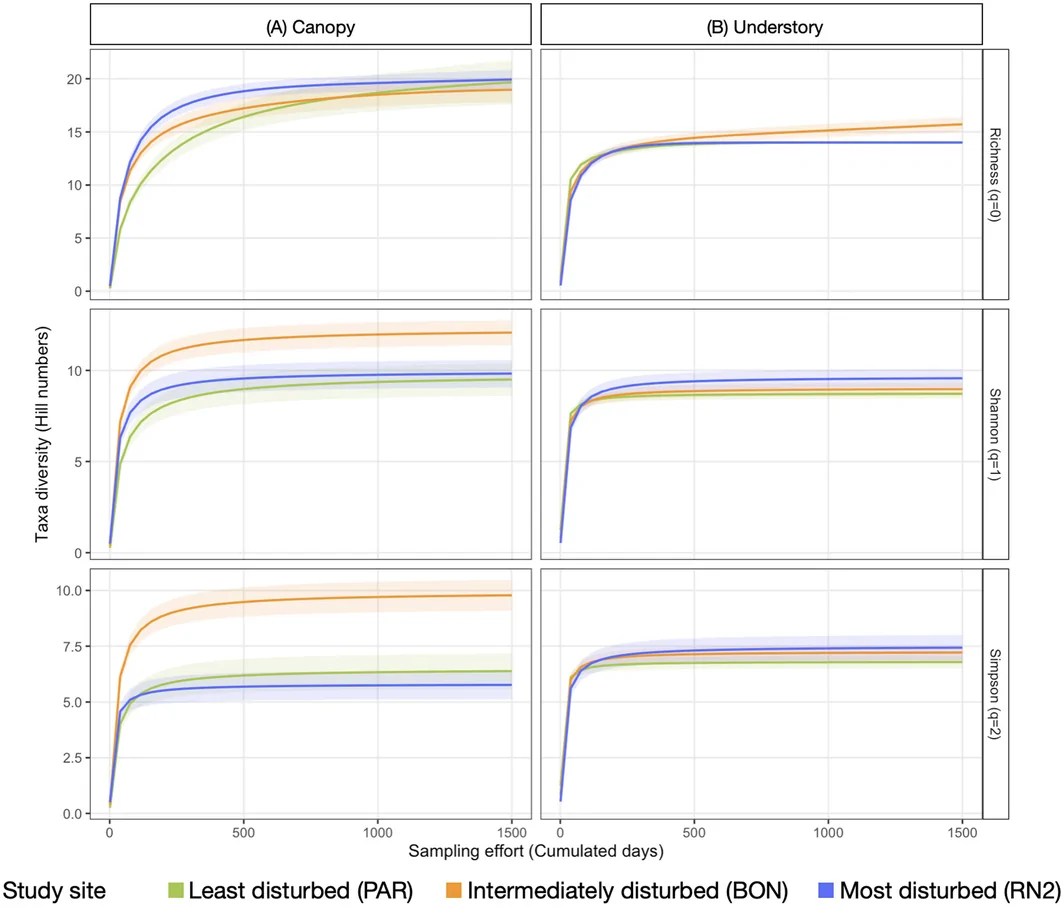
Sample‐size‐based rarefaction curves for Hill numbers (*q* = 0, 1, 2) per stratum (A: canopy, B: understory) and study site, based on the cumulative number of monitoring days. Diversity is represented by taxonomic richness (*q* = 0), Shannon diversity (*q* = 1), and Simpson diversity (*q* = 2). Shaded areas represent the 95% confidence intervals based on 500 bootstrap replicates (Chao et al. 2014, 2016).

##### 2.5.2.2 Frugivores Inventories and Comparison of Visitation Rates Among Sites and Between Strata

We inventoried the detected species per site and strata and calculated the mean RAI per camera. While not accounting for different detection probability among species, it allowed a simple comparison among sites and strata (Figures 5 and 6). We represented (1) the total RAI (all frugivores combined) per site for each stratum (Figure 5); and (2) for each species, the mean RAI per camera per site for each stratum (Figure 6). We then conducted Kruskal–Wallis tests to assess, for all species combined and for each species, if RAIs differed significantly among sites. When significant (*p* < 0.05), we conducted post hoc Dunn's tests with Bonferroni correction to assess the significant differences among pairwise sites.

**FIGURE 5:**
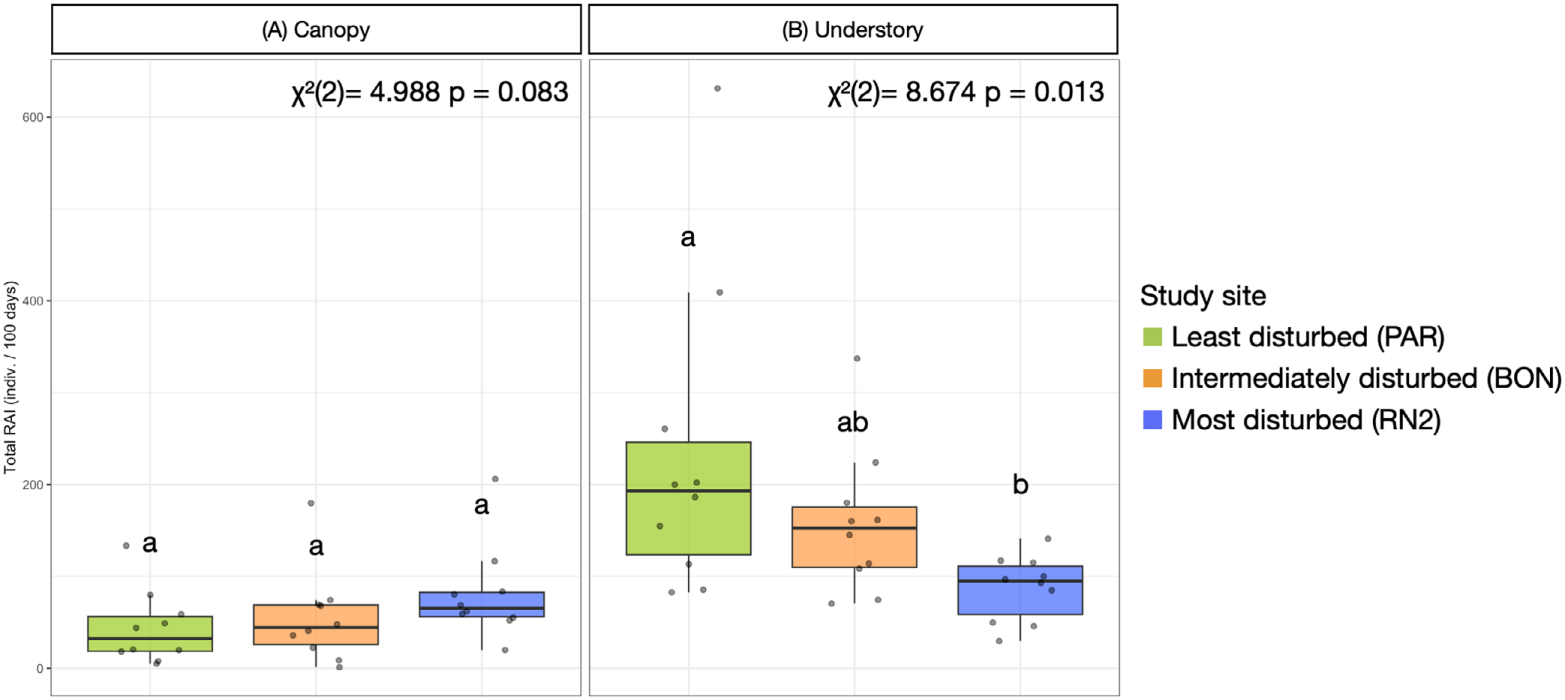
Total visitation rates (RAIs, all frugivores combined) for (A) canopy and (B) understory, per camera trap over the study period (December 2024–May 2025). Each dot represents a camera station. Letters indicate significant differences (Kruskal–Wallis and Dunn's test with Bonferroni correction, *p* < 0.05).

**FIGURE 6:**
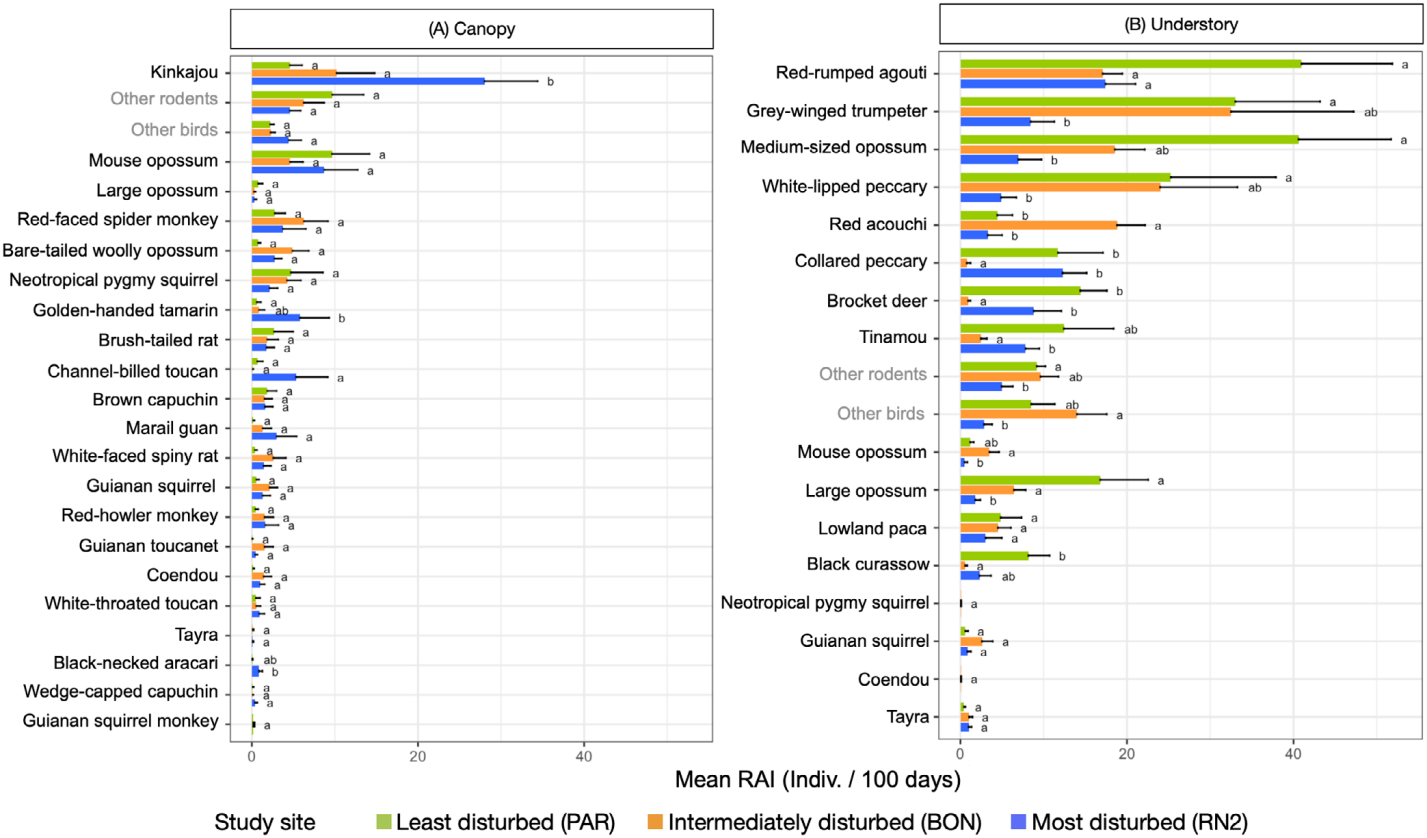
Mean visitation rates (RAIs per species) for (A) canopy and (B) understory, per camera over the study period (December 2024–May 2025). Error bars represent the standard errors. Letters indicate significant differences (Kruskal–Wallis and Dunn's test with Bonferroni correction, *p* < 0.05).

##### 2.5.2.3 Non‐Metric Multidimensional Scaling (NMDS)

To synthesize these observations at the assemblage level, we performed a Non‐Metric Multidimensional Scaling (NMDS) for each stratum, based on Bray–Curtis dissimilarity matrices (Ahumada et al. 2013). Dissimilarities were calculated from species' RAI. To test whether assemblage composition differed significantly among sites, for each stratum, we conducted a PERMANOVA (Permutational Multivariate Analysis of Variance) test using distance matrices. We checked homogeneity of data dispersion within sites. To identify which species contributed most to the observed differences in assemblage composition among sites, we performed a Similarity Percentages analysis (SIMPER) using Bray‐Curtis dissimilarities (Oksanen et al. 2025). This analysis allowed us to rank species based on their contribution to the average dissimilarity between pairs of sites for both the canopy and understory strata.

##### 2.5.2.4 Contribution of Environmental Covariates to Frugivore's Assemblages

To evaluate the influence of environmental factors on frugivore assemblage composition, we performed a Redundancy Analysis (RDA) for each stratum. The response matrices consisted of RAIs for the main frugivore species in our dataset, which were Hellinger‐transformed to account for the high frequency of zeros. The explanatory environmental variables included categorical factors (site identity, tree species) and quantitative environmental descriptors (DBH, elevation, monthly rainfall, tree density, fruit availability, canopy cover) chosen for their possible influence on species visits. Prior to RDA, we assessed multicollinearity using correlation tests with a threshold of |*r*| ≥ 0.60 (Figure S1; Kassambara and Mundt 2020; Wei and Simko 2024). “Tree species” was coded into a binary variable (1 = *V. kwatae*, 0 = *V. michelii*). We only integrated into the RDA uncorrelated variables. Model significance was assessed using a global permutation test for redundancy analyses (999 permutations) (Oksanen et al. 2025). The contribution of each included covariate was determined through marginal permutation tests. When significant, pairwise site's differences were explored using a pairwise PERMANOVA (999 permutations) with Bonferroni correction. Furthermore, we addressed the sample size difference between the two equipped tree species by modeling the impact of tree species identity on overall frugivores RAIs per site, using a linear model including interaction terms between “**s**ite identity” and “strata”, **and “t**ree species” and “strata” (Bates et al. 2015). **We** calculated the estimated marginal means using the emmeans package (Lenth 2025). This approach allowed us to compare sites by averaging over the levels of tree species, and estimate what the overall visitation rates would be if each site had an identical proportion of both tree species. Model validity was confirmed by inspecting residuals with the DHARMa package (Hartig 2024).

## 3 Results

Over the study period, we recorded 2915 arboreal and 6207 terrestrial independent detection events across a total of 5032 cumulated monitoring days in the canopy and 5107 in the understory, with the sampling effort equally distributed among the sites despite occasional camera malfunctions (Table 2).

### 3.1 Comparison of Diversity Indices Among Sites and Strata

Sampling completeness was nearly exhaustive across all study sites and strata, with sample coverage values above 99.68%. Excluding unidentified rodents and birds, 31 fruit‐consumer species were detected (16 arboreal, 10 terrestrial, and 5 semi‐arboreal). All species were detected across all study sites except the Guianan squirrel monkey ( *Saimiri sciureus* ), only detected at the protected site (PAR).

#### 3.1.1 Species Richness

In the canopy, the observed richness reached 20 species in both the protected (PAR) and most disturbed (RN2) sites, and 19 species in the intermediate (BON) site. While RN2 maintained a slightly higher species accumulation rate, all site curves overlapped significantly after 500 days of effort, indicating no statistical difference between them at the end of the study. In the understory, the intermediate site (BON) was the most diverse with 16 species (SC = 99.89%), while both the protected and most disturbed sites reached a plateau at 14 species. The two extra species found at BON were the coendous ( *Coendou prehensilis* and *Sphiggurus melanurus* ) and the neotropical pygmy squirrels ( *Sciurillus pusillus* ), both of which are arboreal or semi‐arboreal and rarely captured in the understory. The 95% CIs for the intermediate site (BON) did not overlap with the protected (PAR) or the most disturbed (RN2). Overall, species richness reached a plateau more rapidly in the understory (100–200 cumulated monitoring days) than in the canopy (> 500 cumulated monitoring days) (Figure 4).

#### 3.1.2 Shannon Diversity

In the canopy, Shannon diversity varied strongly across sites: the intermediate site (BON) exhibited the highest Shannon diversity, followed by the most disturbed site (RN2), while the protected site (PAR) recorded the lowest value. In contrast, in the understory, Shannon diversity was comparable across all study sites, though the most disturbed (RN2) and intermediate (BON) exhibited slightly higher values than the protected site (PAR).

#### 3.1.3 Simpson Diversity

Simpson diversity exhibited patterns consistent with Shannon diversity. In the canopy, the intermediate site (BON) exhibited a significantly higher Simpson diversity compared to the two other sites, which showed lower, nearly identical values with overlapping 95% confidence intervals. In the understory, however, values remained slightly higher in the most disturbed and intermediate sites than in the protected site.

### 3.2 Visitation Rates and Frugivore Inventories Per Site and Stratum

#### 3.2.1 Overall Frugivore Visitation Rates Per Stratum and Sites

In the canopy, overall visitation rates per tree did not vary significantly among sites (*χ* ^2^(2) = 4.988, *p* = 0.083) (Figure 5A). However, there was a trend for higher visitation rates with increasing disturbances, with 43.5 ± 12.5 individuals/100 trap‐days at the protected (PAR), 54.7 ± 16 at the intermediate (BON) and 80.3 ± 16 at the most disturbed (RN2). All sites performed similarly once tree species effects were controlled (*p* > 0.350 for all pairwise comparisons).

In contrast, in the understory, overall visitation rates per tree varied significantly among sites (*χ* ^2^(2) = 8.674, *p* = 0.013) with terrestrial frugivores' visitation rates recorded by our camera traps tending to decrease with increasing disturbances (protected 232.5 ± 53.7/100 trap‐days; intermediate 157.5 ± 25; most disturbed 87.2 ± 11.2), although the only significant pairwise comparison was between the protected and the most disturbed (Dunn's *p* = 0.012; Figure 5B). Moreover, after controlling for tree species effect, significant differences were still detected among sites: specifically, RN2 showed significantly lower visitation rates compared to both the protected site PAR (*p* = 0.004) and the disturbed site BON (*p* = 0.002). No significant differences were detected between BON and PAR (*p* = 0.930).

#### 3.2.2 Individual Species Inventories and Visitation Rates Per Species

The arboreal assemblage visiting *Virola* spp. all sites concerned was mainly constituted of arboreal rodents, kinkajous, small and large opossums (mainly *Marmosa* spp. and bare‐tailed wooly opossums ( *Caluromys philander* )), primates (e.g., golden‐handed tamarin, spider monkey) and toucans (mainly *Ramphastos* spp.) (Figure 6A). A major finding was the dominance of visits of kinkajous at the most disturbed site compared to the two others (*χ* ^2^(2) = 12.320, *p* = 0.002). Regarding primates, while many species remained stable, smaller primates like the tamarin also showed significantly higher activity at the most disturbed site (RN2) compared to the protected site (PAR). Most other arboreal species (toucans, squirrels, larger monkeys) did not show significant differences among sites.

Unlike the canopy, the understory exhibited more differences among sites (Figure 6B). The agouti remained the dominant detected terrestrial frugivore across all three sites with no significant differences in visitation rates among sites (*χ* ^2^(2) = 5.667, *p* = 0.058). At the most disturbed site (RN2), several species showed significantly lower visitation rates compared to the protected site, including trumpeters (*χ* ^2^(2) = 7.021, *p* = 0.030), white‐lipped peccaries (*χ* ^2^(2) = 6.981, *p* = 0.031), and large opossums (*Didelphis* spp.) (*χ* ^2^(2) = 11.346, *p* = 0.003). In contrast, other species such as pacas (*χ* ^2^(2) = 3.358, *p* = 0.187) showed similar visitation rates between the protected and the most disturbed (Cumulated RAIs values per site are reported in Table S2).

### 3.3 Assemblage Structure: Stronger Site‐Specific Differentiation in the Understory Than in the Canopy

According to the NMDS and PERMANOVA analyses, assemblage composition differed significantly among sites in both the understory and the canopy during the study period. In the canopy (stress 0.201), the effect of site identity was significant (*F*_2,27_ = 1.96, *p* = 0.007), explaining 12.7% of the variance (Figure 7A). The protected and intermediate sites showed high similarities, while the most disturbed showed higher differences in assemblage structure (Figure 7). In the understory (stress 0.168), site identity had a stronger significant effect on assemblage structure (*F*_2,27_ = 4.62, *p* = 0.001), explaining 25.5% of the total variance. The three sites only overlapped partially (Figure 7B). The SIMPER analysis revealed that in the canopy, only slight differences were observed between the protected (PAR) and intermediate (BON) sites, which were primarily driven by the visitation rates of bare‐tailed wooly opossums (*p* = 0.001), spider monkeys (*p* = 0.047) and squirrels (*p*‐values ranging from 0.022 to 0.036). The differences between the most disturbed site (RN2) and the intermediate (BON) were characterized by the high contribution of kinkajous (*p* = 0.013) and tamarins (*p* = 0.049). Compared to the protected site (PAR), RN2's dissimilarity was mostly explained by kinkajous (*p* = 0.001), tamarins (*p* = 0.033), and ramphastids, specifically channel‐billed toucans (*p* = 0.040) and black‐necked aracaris (*p* = 0.001).

**FIGURE 7:**
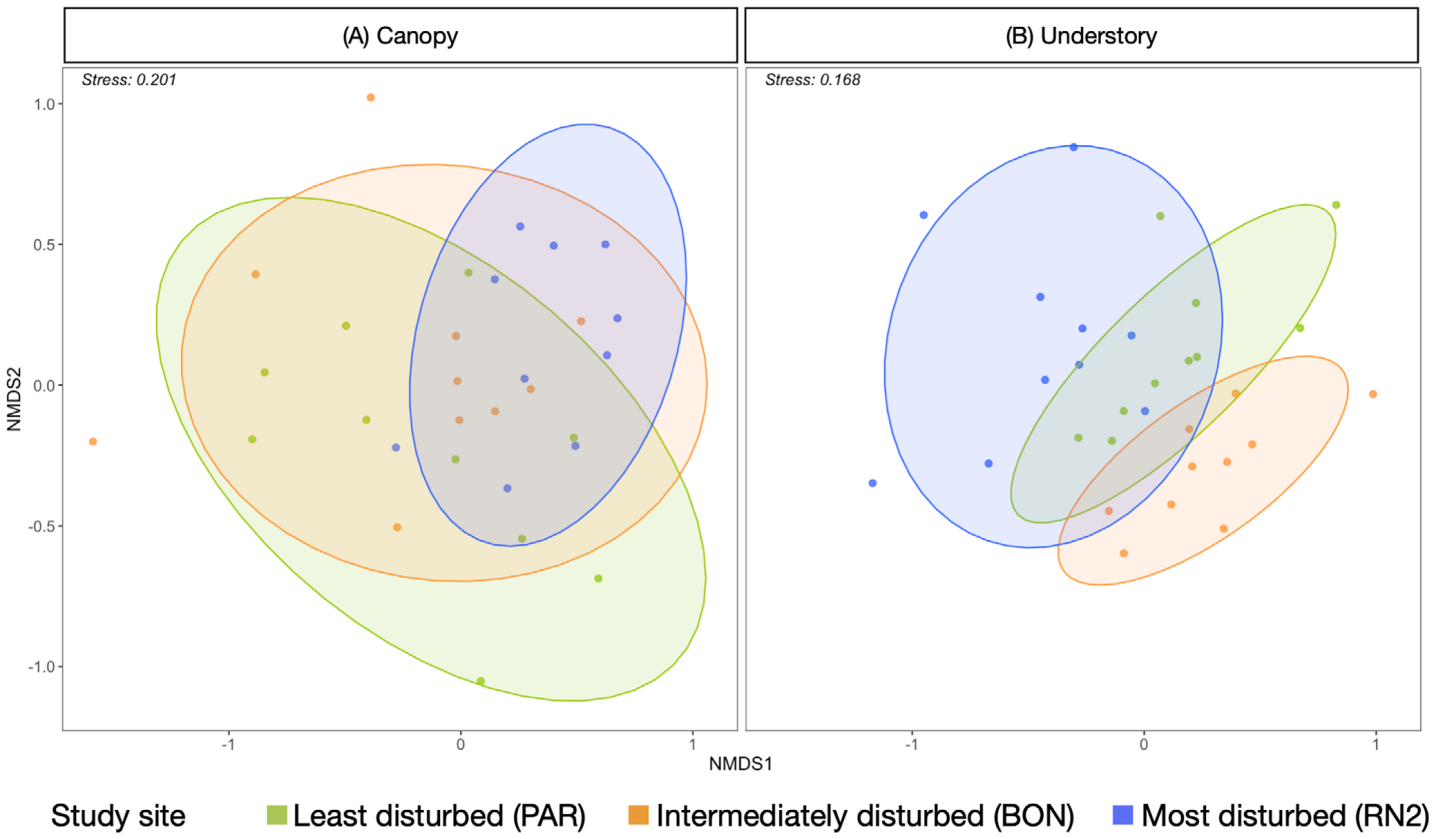
Non‐Metric Multidimensional Scaling (NMDS) ordination based on the Bray–Curtis dissimilarity for assemblage composition. Each point represents a camera trap and is colored according to its study site. Ellipses group cameras from the same site. (A) canopy, (B) understory.

In the understory, no species significantly contributed to any differences among BON and PAR in the understory assemblage. The dissimilarity between the intermediate (BON) and the most disturbed site (RN2) was largely attributed to ungulates and rodents, specifically collared peccaries (*p* = 0.002) and red acouchis (*p* = 0.001). The contrast between the protected site (PAR) and the most disturbed (RN2) was primarily driven by medium and large opossums (*p*‐values ranging from 0.001 to 0.004), agoutis (*p* = 0.012) and black curassows (*p* = 0.036).

### 3.4 Contribution of Environmental Covariates to Frugivore's Assemblages

#### 3.4.1 Arboreal Assemblages Were Partly Explained by Local Habitat Structure

The RDA model for the canopy was significant (*p* = 0.001), indicating that environmental variables structure the arboreal frugivore assemblage (Figure 8A). However, they only explained 3% of assemblage variation (adjusted *R* ^2^ = 0.034). The most influential factor was site identity (*p* = 0.002). DBH (*p* = 0.045), fruit availability (*p* = 0.015) and canopy cover (*p* = 0.016) were also significant predictors. Pairwise's site comparisons revealed that BON and PAR showed no significant difference in assemblage composition (*p*_adj_ = 1.000), whereas the assemblage at the most disturbed site (RN2) was significantly distinct from both the intermediately disturbed (BON) (*p*_adj_ = 0.003) and the protected site (PAR) (*p*_adj_ = 0.018). The other tested factors did not significantly explain assemblage variation during the studied period (rainfall *p* = 0.561, tree density *p* = 0.473, tree species *p* = 0.074). Although non‐significant, large primates' visits (spider monkey, red‐howler monkey and brown capuchin) tend to be related to bigger trees and high canopy cover.

**FIGURE 8:**
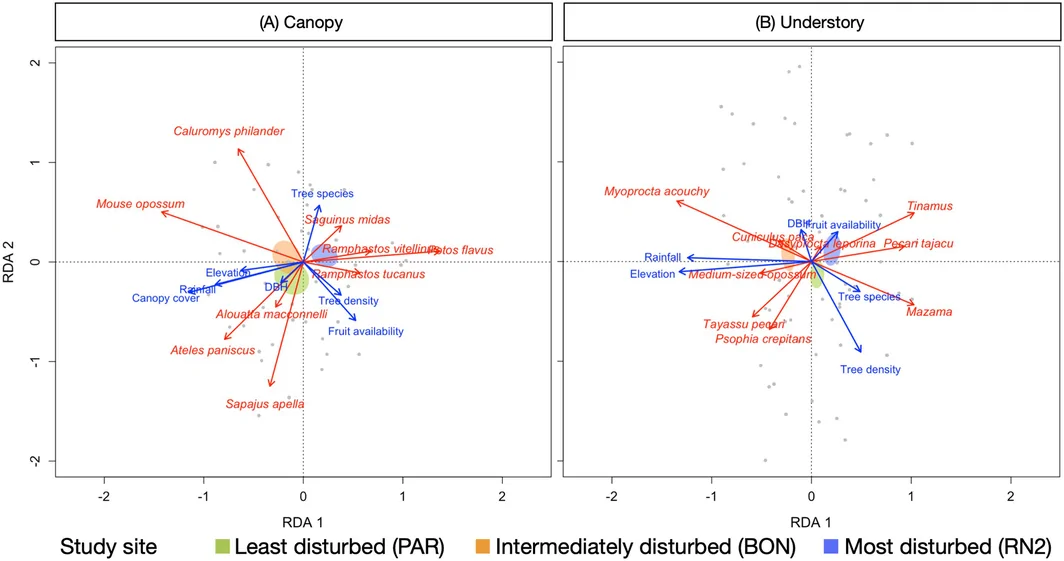
Redundancy analysis (RDA) per stratum (A: canopy, B: understory) using response matrices consisted of Relative Abundance Indices (RAI) for the main frugivores, which were Hellinger‐transformed to account for the high frequency of zeros. Gray points represent the community composition for each station‐month combination. Blue arrows represent environmental explanatory variables; red arrows represent species scores, showing their association with environmental factors and sites. Ellipses indicate 95% confidence intervals for each study site.

#### 3.4.2 Understory: Site‐Dependent Assemblages

The understory model was also globally significant (*p* = 0.001), although it explained a lower overall proportion of variance (adjusted *R* ^2^ = 0.011) compared to the canopy (Figure 8B). Site identity was the only significant predictor for terrestrial frugivores (*p* = 0.003). None of the measured micro‐habitat variables (e.g., DBH, elevation, fruit availability, tree species) significantly influenced the assemblage (*p* > 0.360 for all).

## 4 Discussion

### 4.1 Comparison of Diversity Indices Across Study Sites and Strata

Contrary to our initial predictions, species richness per stratum did not significantly differ among sites, suggesting that even chronically disturbed forests can maintain a high diversity of species. In contrast, Shannon and Simpson indices revealed shifts in canopy assemblage structure; the intermediate site (BON) showed higher index values, reflecting higher assemblage evenness. In contrast, lower values at other sites indicated the dominance of certain species (i.e., the kinkajou at RN2). In the understory, overlapping diversity curves showed no significant differences between sites. However, these diversity indices are misleading in the context of defaunation, as assemblages with similar species diversity can differ greatly in composition and abundances and thus deeply impact ecosystem functioning. Furthermore, several authors showed that the relationship between disturbance history and faunal richness was often counterintuitive; species richness has been observed to remain relatively stable across gradients ranging from undisturbed to heavily disturbed landscapes (Mackey and Currie 2000, 2001; Giacomini and Galetti 2013).

### 4.2 Frugivore Inventories and Visitation Rates

#### 4.2.1 Similar Species Pool Per Stratum but Contrasting Visitation Rates Among Sites

Frugivores visitation rates revealed similar species pools per stratum but variations in individual species' visitation rates among sites. In the canopy, the visitation rates of frugivores all combined remained consistent among sites. However, canopy assemblage was characterized by a few species that seem to increase their activity in more disturbed environments, such as kinkajous, tamarins and channel‐billed toucans. Contrary to our initial assumptions, larger/more sensitive species, such as spider monkeys, did not exhibit lower visitation rates at *Virola* trees with increasing human disturbances, although they were only recorded in 3 of the 10 equipped trees at the most disturbed site (RN2). This result could be explained by the “niche compression” hypothesis (MacArthur and Wilson 1967; Wallace 2008). In well‐preserved habitats, a high diversity of available resources allows frugivores to diversify/share their foraging effort across the landscape. In contrast, in disturbed areas where overall food availability is reduced, species may be forced to concentrate their activity on a limited number of high‐quality resource patches, such as large‐fruited *Virola* trees (Gestich et al. 2022; Gilbert et al. 2022). Consequently, the high visitation rates observed in more impacted sites may not reflect higher population densities, but rather a spatial compression of the niche and an increased attraction factor toward a restricted number of key resources in a disturbed environment, although more research would be needed to test this.

By contrast, the lower visitation rates of a broad range of terrestrial frugivores with increasing human disturbances could reflect the cumulative impact of road‐related disturbances such as roadkills, chemical, noise and light pollutions, as well as increased accessibility, which facilitates hunting and resource extraction (Romero‐Muñoz et al. 2020). Despite a low population density at Saint‐Georges‐de‐l'Oyapock, the use of motorized vehicles by hunters enables deep incursions into the forest via the RN2 road. This likely results in a chronic hunting pressure that may have shaped the observed faunal assemblages: although hunting was not directly measured, we frequently found cartridge cases along the RN2 (Richard‐Hansen and Hansen 1998; Grenand et al. 2002; Richard‐Hansen 2005; Korysko 2019). Trumpeters were less frequent at the most disturbed site, likely due to their sensitivity to human pressure (Richard‐Hansen 2005). The same trend was observed for white‐lipped peccaries, although their lower records during the study period may reflect their nomadic movements rather than local extirpation (Richard‐Hansen et al. 2014; Briceño‐Méndez et al. 2020). Conversely, collared peccaries were more frequently recorded, potentially benefiting from reduced competition (Gottdenker and Bodmer 1998; Romero et al. 2013).

Moreover, the results of the linear model suggest that the decreasing trend in frugivore's visitation rates with increasing disturbances in the understory is not explained by the sample size difference between *V. kwatae* and *V. michelii*.

#### 4.2.2 High Conservation Value of the Secondary Forest (BON) Despite Different Assemblage Structure

Despite its history of logging and mining, the intermediately disturbed site (BON) appeared to retain significant conservation value for most studied species: the consistent presence of sensitive species like spider monkeys, trumpeters and white‐lipped peccaries suggests the persistence of a diverse vertebrate assemblage (Table S2). The presence of such diversity in this secondary forest might be explained by local hunting patterns; near Régina—the closest urban center to BON—hunting pressure is often concentrated along rivers via motorized pirogues rather than within the forest interior (Grenand et al. 2002; Korysko 2019; Parry et al. 2007; Chokkalingam and de Jong 2001). However, given our limited six‐month sampling period, these observations should be considered as a preliminary indicator rather than a definitive assessment of long‐term population stability.

### 4.3 Vertical Stratification of Assemblages and Contribution of Environmental Covariates

Our results reveal a vertical stratification in how frugivore assemblages respond to site‐specific conditions and habitat structure. While both strata showed significant assemblage differences across the gradient, the differentiation was stronger in the understory than in the canopy, suggesting that terrestrial frugivores were more sensitive to site‐scale variations or legacy effects than were arboreal frugivores.

#### 4.3.1 Arboreal Assemblage: The Role of Vegetation Structure

In the canopy, the higher overlap between the protected (PAR) and intermediate (BON) sites suggests a certain resilience or maintained mobility among arboreal frugivores in better‐preserved forests. However, the generally lower visitation rates at the most disturbed site (RN2) suggest that human pressures reshape canopy communities. Even though environmental covariates only explained 3% of the variance, the weak positive correlation between primate visitation rates and larger trees and canopy cover suggests that large arboreal species may selectively visit higher quality habitat patches in disturbed landscapes (Cudney‐Valenzuela et al. 2022). The association of toucans with the most disturbed site further illustrates a shift toward more disturbance‐tolerant or mobile species in impacted areas. However, this result may be interpreted with caution as there is evidence that hunting had negative effects on arboreal frugivore biomass (Scabin and Peres 2021).

#### 4.3.2 Understory: Site‐Specific Factors Beyond Vegetation Structure

The understory assemblage appears to be driven almost exclusively by site identity, with none of the measured micro‐habitat variables providing significant explanatory power. This lack of correlation with local vegetation structure suggests that terrestrial frugivores are influenced by landscape‐scale factors not accounted for in our model. In tropical forests, terrestrial species (notably large rodents, ungulates and bird “game” species) are often the primary targets of hunting, which varies significantly between sites regardless of local forest structure (Korysko 2019; Richard‐Hansen et al. 2019; Scabin and Peres 2021).

#### 4.3.3 Implications for Forest Dynamics and Seed Dispersal

These divergent patterns of assemblage structure have critical implications for forest ecosystem functioning (Lewis 2009). The stronger difference in the understory suggests that terrestrial seed dispersal services (e.g., by large scatter‐hoarding rodents or terrestrial birds like trumpeters) might be more vulnerable to site‐specific defaunation than canopy‐mediated dispersal. As different species perform distinct functional roles, ranging from the long‐distance dispersal of large seeds by primates to the secondary dispersal and by scatter‐hoarding terrestrial rodents, these compositional shifts could lead to significant alterations in forest regeneration patterns (Kirika et al. 2008; Fricke et al. 2025). In disturbed sites along the RN2, where the assemblage is significantly modified, we observed a potential for partial functional compensation, notably through the dominance of the kinkajou ( *Potos flavus* ), an effective and mobile seed disperser (Julien‐Laferrière 2001; Séguigne et al. 2022). However, the loss or reduction of other main dispersers could create a functional gap that disturbance‐tolerant species, such as toucans and kinkajous, can only partially compensate, as they may not fully replace the specific dispersal distances or seed‐handling behaviors of the original frugivore community (Magioli et al. 2021; Fricke et al. 2025).

## 5 Conclusion

This study provides a comparative analysis of arboreal and terrestrial frugivore assemblages across Guianan tropical forest sites subject to varying human disturbance intensities. By monitoring the frugivores visiting *Virola* trees, we observed a consistent species pool across the gradient; however, significant variations emerged in visitation rates. The observed differences varied between strata: while canopy assemblages showed signs of functional resilience, with overall stable visitation rates opportunistic species such as kinkajous and tamarins becoming more frequent in disturbed areas, the understory showed signs of defaunation, with declining visitation rates with increasing disturbances. The different responses of these two vertical groups suggest that terrestrial species may be more sensitive to site‐scale anthropogenic factors, such as road proximity or hunting pressure, which likely outweigh the influence of local forest structure. Our findings highlight that even secondary forests, including those previously impacted by gold mining and logging, retain a highly diverse pool of fruit consumers. Although their assemblages are restructured compared to protected areas, these human‐modified landscapes remain vital reservoirs of biodiversity and essential components for maintaining forest resilience and seed dispersal services (Kocher et al. 2017; Da Silva Batista et al. 2025).

## Author Contributions

**Raphaëlle Abensur:** formal analysis (equal), investigation (equal), writing – original draft (equal), writing – review and editing (equal). **Opale Coutant:** investigation (equal), writing – review and editing (equal). **Pierre Gloaguen:** investigation (equal), writing – review and editing (equal). **Malika Gottstein:** writing – review and editing (equal). **Coraline Pelon:** investigation (supporting). **Pierre‐Michel Forget:** conceptualization (equal), funding acquisition (equal), investigation (equal), supervision (equal), writing – review and editing (equal). **Eric Guilbert:** conceptualization (equal), funding acquisition (equal), investigation (equal), writing – review and editing (equal). **Christophe Baltzinger:** conceptualization (equal), funding acquisition (equal), investigation (equal), writing – review and editing (equal).

## Funding

This work was supported by the Office Français de la Biodiversité, Fondation Crédit Mutuel Alliance Fédérale, and Labex DRIIHM.

## Conflicts of Interest

The authors declare no conflicts of interest.

## Supporting information


**Figure S1:** Pearson correlation matrices of environmental variables for (A) canopy and (B) understory strata.
**Table S1:** Spatial autocorrelation analysis (Mantel tests) of frugivore community composition across sites and strata during the wet season.
**Table S2:** Inventory of detected species and Cumulated Relative Abundance Index (RAI) per species for each study site during December 2024 and May 2025 (10 camera traps per stratum per site).

## Data Availability

The data used in this project are available on the database “Effect of anthropogenic activities on wildlife diversity in French Guiana”, hosted on the Agouti platform (http://agouti.eu/). Specific analyzed datasets and R script are available on Zenodo, https://doi.org/10.5281/zenodo.22029095.
