# Supplementary material for: Arboreal and Terrestrial Frugivore Vertebrate Assemblage Diversity and Composition Vary With Disturbance Histories in Guianan Forests
Source: Ecol Evol. 2026 Sep 28;16(10):e74374. doi: 10.1002/ece3.74374 (PMC13620221; doi:10.1002/ece3.74374)
Supplement: Supplementary file 1 [file ECE3-16-e74374-s001.docx]

SUPPLEMENTARY MATERIAL

Table S1. Spatial autocorrelation analysis (Mantel tests) of frugivore community composition across sites and strata during the wet season. Results show the correlation between ecological and geographical distances based on 999 permutations.

Table S2. Inventory of detected species and Cumulated Relative Abundance Index (RAI) per species for each study site during December 2024 and May 2025 (10 camera traps per stratum per site). Arboreality: C = Canopy, U = Understory. Activity: D = Diurnal DN = Diurnal & Nocturnal, N = Nocturnal (Emmons & Feer, 1997). Role: SD = Seed Disperser, SD/SP = Seed Disperser and/or Predator SP = Seed Predator (Théry et al., 1992; Gayot et al., 2004; Boissier et al., 2020). Diet: F = Frugivore, H = Herbivore, O = Omnivore, I = Insectivore (Feer et al., 2001). IUCN status : NT = Near threatened LC = Least Concern VU = Vulnerable DD = Data Deficient (INPN) (Feer et al., 2001).

 Figure S1. Pearson correlation matrices of environmental variables for (A) Canopy and (B) Understory strata.
